# Testing the Fitness Consequences of the Thermoregulatory and Parental Care Models for the Origin of Endothermy

**DOI:** 10.1371/journal.pone.0037069

**Published:** 2012-05-14

**Authors:** Sabrina Clavijo-Baque, Francisco Bozinovic

**Affiliations:** Departamento de Ecología, Facultad de Ciencias Biológicas, Pontificia Universidad Católica de Chile, Santiago, Chile; University of Regina, Canada

## Abstract

The origin of endothermy is a puzzling phenomenon in the evolution of vertebrates. To address this issue several explicative models have been proposed. The main models proposed for the origin of endothermy are the aerobic capacity, the thermoregulatory and the parental care models. Our main proposal is that to compare the alternative models, a critical aspect is to determine how strongly natural selection was influenced by body temperature, and basal and maximum metabolic rates during the evolution of endothermy. We evaluate these relationships in the context of three main hypotheses aimed at explaining the evolution of endothermy, namely the parental care hypothesis and two hypotheses related to the thermoregulatory model (thermogenic capacity and higher body temperature models). We used data on basal and maximum metabolic rates and body temperature from 17 rodent populations, and used intrinsic population growth rate (*R_max_*) as a global proxy of fitness. We found greater support for the thermogenic capacity model of the thermoregulatory model. In other words, greater thermogenic capacity is associated with increased fitness in rodent populations. To our knowledge, this is the first test of the fitness consequences of the thermoregulatory and parental care models for the origin of endothermy.

## Introduction

Continuous endothermy is an exclusive feature of birds and mammals, although endothermic traits are present in several groups of plants, invertebrates, and other vertebrates [1]. Strictly speaking, endothermy is the maintenance of high and constant body temperatures (T_b_) through the production and conservation of metabolic heat [2]. Birds and mammals may produce heat through internal organs such as brain, liver, heart, kidneys and gut, while in other endothermic organisms heat generation occurs only through muscular contraction [3], [4]. In comparison to ectotherms, mammals and birds exhibit a higher T_b_, because they maintain high metabolic rates and exhibit low thermal conductance [1], [2], [5]–[7]. One major benefit of continuous endothermy (hereafter endothermy) is independence from the environment [8], which could account for the relative number and diversity of mammal and avian species in climatically extreme environments [9]. Moreover, endothermic organisms can sustain high levels of activity due to their high capacity for aerobic metabolism. This has important ecological benefits such as the ability to escape from predators or to search for food under a wider range of environmental conditions than ectotherms [10].

Endothermic organisms have high daily costs, for example basal (BMR) or resting metabolic rates (RMR) nearly 20 times higher than metabolic rates of reptiles of similar body size [11], [12]. As a consequence, birds and mammals spend about 30% of their total energy budget on maintenance [1]. Understanding the evolutionary history of these thermal adaptations and the high costs of going from an ectothermic condition to the extant endothermic condition, has been an elusive and controversial topic [13], [14]. The evolution of endothermy, while one of the most important evolutionary steps in vertebrate history, is a puzzling evolutionary event [15].

Several competing hypotheses have been suggested to explain the evolution of endothermy, namely the aerobic capacity model [6], the thermoregulatory model [8], [16] and the parental care model [5], [17]. The aerobic capacity model posits that natural selection favoured sustained activity, a condition related to aerobic capacity during exercise. Aerobic capacity is usually measured as maximum rate of oxygen consumption, a proxy for maximum metabolic rate (MMR). Assuming a structural coupling between MMR and RMR, directional selection on MMR would have generated a correlated response in RMR [6]. The aerobic capacity model assumes that MMR and BMR are heritable and also genetically correlated [18]–[22].

The thermoregulatory model comprises several hypotheses which assume body temperature was the target of natural selection [16], [23]. In one scenario, the thermogenic capacity model, natural selection acted directly on T_b_, but only after selection for the ability to maintain stable T_b_ under different environmental conditions [8]. When proto-mammals became nocturnal their thermal adaptation allowed them to expand their thermal niche, with the resulting colonization of new environments [8]. After this, they returned to diurnal activity, with the consequent increase in metabolic rate and T_b_ [15], [24].

Alternatively, one of the first hypotheses proposed for the origin of endothermy was the adaptation to higher T_b_ values [25] to maximize performance. For example, enzymatic reactions are maximal at particular temperatures, and proto-endotherms probably had high temperature set points, for which increases in T_b_ were selected [25]. We call this version of the thermoregulatory model the “higher body temperature model”. Experimental tests attempting to increase T_b_ in reptiles by increasing metabolic rate have failed to support this higher temperature model. In particular, tripling or even quadrupling the standard metabolic rate (SMR) of experimental subjects during digestion results in an increase in T_b_ of less than 1°C [7]. However, it has recently been suggested that the thermoregulatory profits of an increased RMR could play an important role in the “aerobic capacity” scenario [24].

Finally, the parental care model postulates that the increase in BMR was a by-product of natural selection for parental care [17]. Parental care entails high activity, higher daily energy expenditure (DEE), larger internal organs and, ultimately, a high BMR. Koteja [1] argued that selection on parental care–which increases juvenile survival relative to adults–would be strong (but see [26]). In contrast, the parental care model proposed by Farmer [5] is linked to the thermoregulatory hypothesis. Farmer’s model [5] posits that natural selection acted on incubation temperature to increase developmental stability with a consequent increase in hatchling growth rate. Thus, Farmer’s parental care based hypothesis is not necessarily distinct from the thermoregulatory model, as it relies on an additional benefit of high T_b_ [1], [27].

Several studies have tested the various models for the origin of endothermy by analyzing assumptions of the models such as the relationship between BMR and MMR [7], [18], [19], [23]. Nevertheless, a key challenge is to determine the links between each factor that has been proposed as a major functional determinant in the evolution of endothermy and fitness. To resolve this problem it is necessary to identify the functional traits that are important to proto-endotherms and the strength of selection to which they were subjected. Clearly, this information is not available, but we suggest that evidence gathered from extant populations may shed light on what happened in the past. Knowing how energetic traits are associated with fitness in the present is a first step towards understanding the evolution of endothermy [22]. Furthermore, apart from the relationship between the target trait and fitness, each model for the evolution of endothermy assumes different causal relationships among metabolic traits, which have not yet been analyzed together. Consequently, the aim of our study was to test the thermoregulatory and Koteja’s parental care models for the origin of endothermy. To this end, we carried out a cause-effect analysis of the relative effects of BMR, MMR and T_b_ on fitness. This allowed us to infer the target of natural selection on endothermy in extant populations. To do this we estimated fitness as the intrinsic growth population rate (*R_max_*) reported for different species’ populations, which includes processes of both reproduction and survival. The strength of association was evaluated using Structural Equation Modeling (SEM; also referred to as path modeling). These statistical models represent a series of hypothesized cause-effect relationships, which can be viewed as a composite hypothesis concerning patterns of statistical dependencies [28]. Once a hypothesis has been proposed, it can be tested against empirical data using SEM. Then, it is possible to construct a set of candidate models which represent different theoretical models, or competing hypotheses, and compare their viability given the available data. The relative strength of each hypothesis was evaluated using an information criterion such as the Bayesian Information Criterion (BIC).

### Predicitons of Endothermy Models

**Figure 1:**
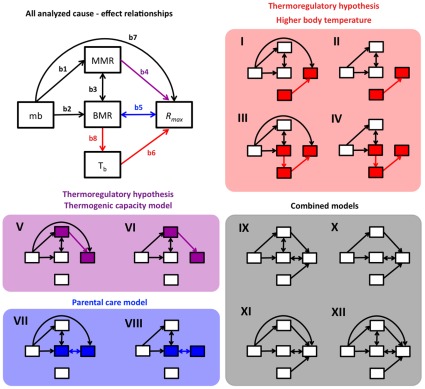
Path diagrams of the evaluated causal models. The chart without shading shows all considered cause-effect relationships, colored arrows are relationships related to the tested models for endothermy while black arrows are unrelated relationships. Arrows have their corresponding number and variables. The shaded charts show the 12 models evaluated in this work, highlighting which endothermy models are represented and the involved variables. mb = body mass, BMR = basal metabolic rate, MMR = maximum metabolic rate, *R_max_* = intrinsic population growth rate; T_b_ = body temperature. Note that for testing Koteja’s parental care model, path b5 is a correlation (indicated by bidirectional arrows), because the relationship between both variables is mediated by parental care, as proposed by the model.

All proposed models for the origin of endothermy, unfortunately, are verbal and there is no mathematical representation of them. This makes it difficult to test them rigorously (Angilleta, 2010). Hence, we translate the verbal models to mathematical ones (path models) including the assumptions and relationships which represent the proposed mechanism for the origin of endothermy (Figure 1, Table 1). First, the higher body temperature model posits that selection favoured a higher T_b_s [25]. Therefore, an increase in this variable should be associated with an increase in *R_max_*. Thus, we expected to find that path b6 was significantly different from zero (Figure 1). The responsible mechanism to increase T_b_ is not clear; some authors suggest that T_b_ increases could be due to increase in BMR [2]. Therefore, we represented this relationship with path b8, from BMR to T_b_ (Figure 1, Table 1). However, experimental evidence suggests that significant increases in BMR do not increase T_b_ [7]; furthermore, increases in T_b_ may be because of changes in conductance [13]. In this case, path b8 could not be considered to test the higher body temperature model.

**Table 1:** Models tested in this work are presented as structural equations (for graphical representation see Figure 2).

Model		Structural equation
Thermoregulatory: Higher Tb	I	*R_max_* = b6 T_b_+b7 mb; BMR = b3 MMR+b2 mb; MMR = b1 mb+b3 BMR
Thermoregulatory: Higher Tb	II	*R_max_* = b6 T_b_; BMR = b3 MMR+b2 mb; MMR = b1 mb+b3 BMR
Thermoregulatory: Higher Tb	III	*R_max_* = b6 T_b_+b7 mb; BMR = b3 MMR+b2 mb; MMR = b1 mb+b3 BMR; T_b_ = b8 BMR
Thermoregulatory: Higher Tb	IV	*R_max_* = b6 T_b_; BMR = b3 MMR+b2 mb; MMR = b1 mb+b3 BMR; T_b_ = b8 BMR
Thermoregulatory: thermogenic capacity	V	*R_max_* = b4 MMR+b7 mb; BMR = b3 MMR+b2 mb; MMR = b1 mb+b3 BMR
Thermoregulatory: thermogenic capacity	VI	*R_max_* = b4 MMR; BMR = b3 MMR+b2 mb; MMR = b1 mb+b3 BMR
Parental Care	VII	*R_max_* = b5 BMR+b7 mb; BMR = b5 *R_max_*+b3 MMR+b2 mb; MMR = b1 mb+b3 BMR
Parental Care	VIII	*R_max_* = b5 BMR; BMR = b5 *R_max_*+b3 MMR+b2 mb; MMR = b1 mb+b3 BMR
Combined models	IX	*R_max_* = b4 MMR+b5 BMR+b6 T_b_+b7 mb; BMR = b3 MMR+b2 mb+b5 *R_max_*; MMR = b1 mb+b3 BMR
Combined models	X	*R_max_* = b4 MMR+b5 BMR+b6 T_b_; BMR = b2 mb+b5 *R_max_*; MMR = b1 mb
Combined models	XI	*R_max_* = b4 MMR+b5 BMR+b6 T_b_+b7 mb; BMR = b2 mb; MMR = b1 mb
Combined models	XII	*R_max_* = b4 MMR+b5 BMR+b6 T_b_; BMR = b3 MMR+b2 mb; MMR = b1 mb+b3 BMR

mb = body mass, MMR = maximum metabolic rate, BMR = basal metabolic rate, *R_max_* = intrinsic population growth rate; T_b_ = body temperature.

The thermogenic capacity model says that selection acted on the capacity to maintain constant body temperature, during cold exposure [2]. Then, increases in MMR should be positively related to an increase in fitness, represented by path 4 (Figure 1). The higher body temperature model further assumes that T_b_ increases, whereas the thermogenic capacity model does not explicitly explain how higher T_b_ was achieved [25]. So, path b8 was not included in path models representing the thermogenic capacity model (Figure 1).

Koteja’s parental care model is similar to the aerobic capacity model in that it suggests selection favoured increased locomotor activity [17]. However, the main difference between the parental care and aerobic capacity model is that the parental model suggests this increase in locomotor capacity may have been necessary for the evolution of enhanced parental care [17]. Additionally, the parental care model posits that BMR increased as a by-product of selection for enhanced parental care [17]. Therefore, increasing BMR should be correlated with increasing *R_max_* (b5) (Figure 1). Path b5 has a double headed arrow, meaning that this relationship in our model is correlational [29] and may be mediated by other variables not included in our data set (i.e. parental care and DEE).

Finally, the hypotheses are not mutually exclusive [22], [24], therefore, we also tested some combined models which have the relevant relationships for higher body temperature, thermogenic capacity and parental care models (Figure 1). We further included paths to account for the relationship between metabolic rates (BMR and MMR) and mb (b1 and b2, respectively), BMR and MMR (b3), and mb and *R_max_* (b7). The relationship between MMR and BMR is an assumption of the aerobic capacity model, which we did not test; however, we consider that it is accurately represented in our models because the genetic correlation between MMR and BMR has been reported for several species [18]–[22]. The relationship between mb and *R_max_* (b7) has been reported previously for mammals [30], so we include it in our models.

## Results

We found no phylogenetic signal linked to any variables tested (Table 2), thus we did not correct our data for phylogenetic trends.

**Table 2:** Estimation of phylogenetic signal in physiological and population level variables using the K and λ parameters.

Variable	K*	λ*
mb	0.098	0.000
BMR	0.095	0.000
MMR	0.105	0.000
*R_max_*	0.282	0.000
T_b_	0.275	0.676

* Parameters close to zero imply no phylogenetic signal. mb = body mass, BMR = basal metabolic rate, MMR = maximum metabolic rate, *R_max_* = intrinsic population growth rate, T_b_ = body temperature.

**Figure 2:**
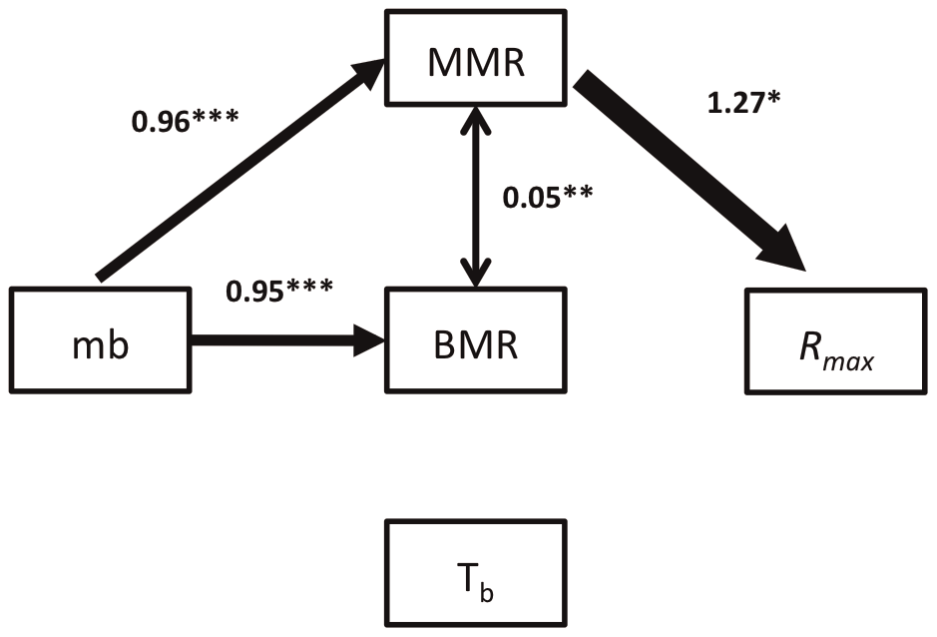
Schematic of thermogenic capacity model (model VI), the best fitting model. The parameter estimated for each path and their associated probability are indicated above arrows (*** = *P*<0.001 with ML, ** = *P*<0.05 with ML, * = distinct from 0 based on bootstrap). The arrows’ thickness is proportional to the estimated path’s coefficient. mb = body mass, BMR = basal metabolic rate, MMR = maximum metabolic rate, *R_max_* = intrinsic population growth rate; T_b_ = body temperature.

The best fit to the available data was attained with model VI (Table 3), which only included a path associated with the thermogenic capacity model (Figure 2). This model explained 18% of the variance in *R_max_* whereas the other related model with thermogenic capacity (model V), explained 23% of the variance (Table 3), but with fewer degrees of freedom (Figure 1; Table 3). Degrees of freedom decrease when the number of parameters estimated (i.e. paths included in the model) increase, while the explained variance increases with number of parameters in the model [29].

**Table 3:** Indices used for model selection and percentage of variance explained for the response variable.

Model	χ^2^	d.f.	*P*	BIC	RMSEA	*r^2^*
Model VI	3.30	8	0.914	−19.3	0	0.18
Model V	2.28	7	0.943	−17.5	0	0.23
Model VII	6.42	8	0.60	−16.3	0	0.00
Model II	6.33	8	0.61	−16.3	0	0.01
Model I	4.13	7	0.77	−15.7	0	0.14
Model XII	2.75	6	0.84	−14.3	0	0.20
Model VII	3.03	6	0.81	−14	0	0.20
Model IX	1.29	5	0.94	−12.9	0	0.27
Model IV	6.14	6	0.41	−10.9	0	0.01
Model III	3.94	5	0.56	−10.2	0	0.14
Model X	11.08	7	0.14	−8.9	0.19	0.20
Model XI	10.65	6	0.09	−6.4	0.22	0,26

χ^2^ = Chi square value and associated probability level (where p>0.05 indicates the model could not be rejected); BIC = Bayesian Information Criterion (lower values indicate a better model), RMSEA = root means square error approximation (<0.05 is interpreted as adequate fit; Shipley, 2000), *r^2^* = explained variance in *R_max_*.

Models including paths associated with the higher body temperature model (I-IV) and the parental care model (VII-VIII) explained a relatively small proportion of variances, and yielded higher values of BIC (Table 3 and 4).

**Table 4:** Structural equations for the most representative theoretical models.

Model	Structural equation*
Model VI	*R_max_* = 1.27 MMR ^(*P* = 0.059)^ *; BMR = 0.95 mb ^(*P* = 0.000)^+0.05 MMR ^(*P* = 0.026)^; MMR = 0.96 mb ^(*P* = 0.000)^+0.05 BMR ^(*P* = 0.026)^
Model V	*R_max_* = 1.27 MMR ^(*P* = 0.135)^−0.87 mb ^(*P* = 0. 305)^; BMR = 0.95 mb ^(*P* = 0.000)^+0.05 MMR ^(*P* = 0.026)^; MMR = 0.96 mb ^(*P* = 0.000)^+0.05 BMR^(*P* = .0026)^
Model IV	*R_max_* = −0.102 T_b_^ (*P* = 0.679)^; BMR = 0.95 mb ^(*P* = 0.000)^+0.05 MMR; T_b_ = 0.101 BMR ^(*P* = 0.660)^; MMR = 0.96 mb+0.05 BMR
Model VII	*R_max_* = 0.018 BMR^ (*P* = 0.018)^; BMR = 0.95 mb ^(*P* = 0.000)^+0.05 MMR^(*P* = 0.05)^+0.018 *R_max_* ^(*P* = 0.018)^; MMR = 0.96 mb ^(*P* = 0.000)^+0.05 BMR^(*P* = 0.05)^

* For each equation all of the variables included and causally connected with other variables present in the model are shown. The number in front of the variable’s name indicates the path’s parameter and the p value for the path, estimated using ML, is shown in parenthesis. mb = body mass, BMR = basal metabolic rate, MMR = maximum metabolic rate, *R_max_* = intrinsic population growth rate, T_b_ = body temperature.

The thermogenic capacity model (model VI) had a path coefficient for b4 (MMR to *R_max_*) which was marginally significant using maximum likelihood (ML) (Table 4), and significant based on bootstrapping. We compared strength of the cause-effect relationships by comparing the estimated path coefficients in our path models. In this sense, path b3, from MMR to BMR, had lower estimate than the other paths in model VI. Nevertheless, b3 was implicated as an important link because it was significant in the best model, and all models that did not include this path fit the data poorly (Table 3). In contrast, path b7 (mb to *R_max_*) was not significant in any model; moreover, models that included this path were inferior (BIC>−16). Finally, path b8 (BMR to T_b_) was poorly supported (Table 3).

## Discussion

The thermogenic capacity model, represented by model VI, best describes the data and assumes a direct functional relationship between MMR and fitness (Table 3; Figure 2). Our path analysis revealed that the best fit to the data was obtained with a model representing the thermogenic capacity hypothesis. This is not the first time that the relationship between metabolic rates and fitness, or some of its components (survival and reproduction), have been tested [31]. Nonetheless, until now there has been insufficient data regarding the strength of natural selection on metabolic rates to understand the probability of it explaining the origin of endothermy [22].

Our results suggest that MMR, not BMR, determines the relationship between generation time and reproductive rate, as previously suggested [32]–[34]. An advantage of our work is that we tested causal relationships, with mass affecting both metabolic rates. Further agreement with previous information is the observation that both mass-independent BMR and MMR were correlated [35]–[39]. This connection was supported by the observation that path b3 was significantly distinct from zero (Table 4).

The best fitting model was the thermogenic capacity model (model VI), given both ML and bootstrap analyses. This model represents the thermogenic capacity model, and path b4 (from MMR to *R_max_*) was significant and positive. Even though the percentage of *R_max_* explained was only 18%, this is considerable given that *R_max_* is a global estimate which is also influenced by several other factors, such as environmental productivity, life cycle and phylogeny [34].

While our analysis may be criticized on the basis of a small sample size (we only had access to 17 species for which data for all parameters were available), it is important to note that Model VI fit (see Table 3) with high statistical power (RMSEA index close to zero) [40]. Furthermore, we can differentiate the thermogenic capacity model (model VI) from all other models using BIC, since it allows us to discriminate between competing models penalizing for small sample size. Although the data we used come from populations belonging to a derived group among mammals, this is, as far as we know, the first time that a global estimator of fitness, like *R_max_*, has been used to examine the fitness consequences for the origin of endothermy.

Also noteworthy was that path b7 did not occur in our best model. While this relationship (from mb to *R_max_*) has been reported for mammals [30], it was not significant. Likely, to be due to the fact we studied rodent populations with a smaller range of body sizes. Moreover, b6 (T_b_ to *R_max_*) was poorly supported (Table 3) and any model that contained only this relationship poorly described the data, explaining less than 1% of the variance in *R_max_* (Table 3). Perhaps, it was not significant because T_b_ varies too little. As a result, it is not possible to reject the higher temperature model; rather, it has the poorest experimental evidence [7], an absence of fossil evidence [14], and a lack of statistical support from natural populations. Taken together, these findings imply that endothermy arose as a mechanism to expand thermogenic capacity.

Finally, since metabolic rates set the pace of life, measurements and analysis of their variability and evolution have been, and continue to be, of paramount importance to several contemporary evolutionary and ecological theories, which attempt to link animal energetics to traits such as species richness, species distribution, life-history strategies and evolutionary processes. Now, based on a bioenergetics approach, we provide support for the thermogenic capacity model for the origin of endothermy. Clearly, this assumes that the processes currently operating were similar to those that operated in the past [9], [41] and that inter-specific rodent’s variability represents at least part of the proto-endotherms variability. In this sense, similar studies on reptile and bird populations are still needed to evaluate the generality of our results. It is important to note that we did not test the aerobic capacity model [42].

## Materials and Methods

### Source of Data

We considered the following physiological variables: BMR, T_b_, and MMR. Body mass (mb) was also included through its effects on metabolic rates [2]. We used MMR obtained during exposure to cold temperatures and in He-O_2_ atmospheres [43], [44].

To perform path analyses we selected all species of rodents where data were available on metabolic rates measured in the same individuals, together with the corresponding data of population dynamics. We conducted path analysis [29] with data obtained for 17 rodent species from North and South America, Australia and Europe, covering a size range from 6 g to 900 g (for more details see Table S1). We used inter-specific comparisons assuming that the interspecific variation represents intraspecific variability in physiological traits evolved through natural selection. In other words, after several generations of positive selection acting on these traits, it is more likely that any extant species has the physiological variability comparable to proto-endotherms variability. Therefore, we preferred inter-specific over intra-specific comparisons. Physiological data were taken from the literature, and we chose articles where all measurements came from the same set of individuals.

Although interesting, most of the studies which analyzed relationships among metabolic rates and fitness used proxies of fitness based on only one of its components [31], [45]. We used *R_max_* as a proxy of fitness, which is a more inclusive measurement since it includes both reproduction and survival. In short, *R_max_* is an estimate of how long an average individual lives and how many descendants it leaves in the population [46]. In spite of its accuracy, it is not frequently used since estimations of *R_max_* require several years of population data. Data on intrinsic population growth rates (*R_max_*) were usually obtained from studies different from those reporting physiological measurements. However, we chose reports of *R_max_* obtained from populations inhabiting geographically or environmental similar habitats relative to those of studies used as sources of metabolic variables. We also selected this procedure to avoid noise and variation between populations owing to local adaptation. Whenever direct estimates of *R_max_* were not available, we calculated *R_max_* estimates from data on time series through cubic splines to avoid problems of convergence [47]. This method finds a different equation for every pair of adjacent points, and selects the equations such that the overall curve is smooth [48].

### Phylogenetic analysis

To examine whether evolutionary relationships among species could confound our analysis [49]–[51], we quantified the phylogenetic signal associated with each variable [52]. To do so, we first built a phylogeny of the species based on DNA sequences for interphotoreceptor retinoid-binding (IRPB), which were gathered from GenBank®. When sequences for target species were unavailable, we employed sequences from closely related species based on previous and unrelated phylogenetic analysis (Table S2). Sequence alignment was conducted online with a Muscle Alignment and “A la carte” mode [53]; maximum-likelihood searches were carried out with PhyML [54]. The phylogenetic tree used to estimate phylogenetic signal is shown in Figure S1. This tree is congruent with most previously published phylogenetic relationships across the main families of Rodentia [55]–[57]. We then tested for phylogenetic signal on all variables using the packages of Picante and Geiger for the R platform [58]–[60]. We calculated the “K” and “λ” parameters [49]. Since we did not find phylogenetic signal linked to any of the examined variables (Table 2), we did not correct our data for phylogenetic relatedness.

### Modeling

We used SEM to evaluate functional relationships between fitness and the physiological traits that have been proposed as critical targets of natural selection in the alternative models for the evolution of endothermy. This method allows the evaluation of a cause-effect relationship between variables, and also allows us to contrast theoretical models [28]. In SEM, the relationships (arrows) are described by parameters (path coefficients) that indicate the magnitude of the effect (which can be direct or indirect, or a combination) of the independent variables on the dependent variables, (see [28]). We conducted a path analysis with the aim of testing some critical co-variations predicted by alternative models for the evolution of endothermy (Figure 1) [29].

The variables were log transformed when it was necessary to meet normality assumptions. Additionally, all variables were standardized using the correlation matrix in path analyses so that all estimated coefficients could be compared. We used Maximum-likelihood (ML) tools to estimate path coefficients and their associated probability values in structural equations [29]. Afterwards, we used bootstrapping to calculate confidence intervals associated with each path coefficient. This approach is powerful for examining small data bases and provides an adequate evaluation of evolutionary and ecological hypotheses [61]. We used “sem” and “boot” packages in Program R [62], [63]. Model selection was conducted using χ^2^ (P>0.05 model could not be rejected), BIC (comparatively lower values indicate a better model), index root means square error approximation (RMSEA, near to 0 is considered a good fit), and variance explained to determine that the best fitted model was accurate to explain the data [29], [61]. We used BIC since it allows us to discriminate between competing models when penalizing for small sample size [29], [61].

## Supporting Information

Figure S1 Phylogenetic tree resulting from the maximum-likelihood analysis of the IRPB gene sequences of 17 rodent species and 1 outgroup (*Lepus crawshayi*).
(TIF)

Table S1 Alphabetical listing of metabolic and population data compiled for 17 species of rodents with references.
(DOCX)

Table S2 IRBP gene sequences used in phylogenetic reconstruction and its GenBank code.
(DOCX)
