# Supplementary figures and images for: Testing the Fitness Consequences of the Thermoregulatory and Parental Care Models for the Origin of Endothermy
Source: PLoS One. 2012 May 14;7(5):e37069. doi: 10.1371/journal.pone.0037069 (PMC3351390; doi:10.1371/journal.pone.0037069)

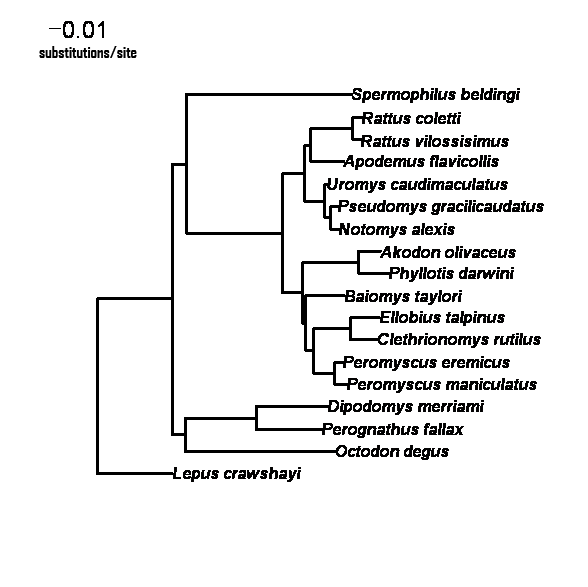

Supplement: Figure S1 — Phylogenetic tree resulting from the maximum-likelihood analysis of the IRPB gene sequences of 17 rodent species and 1 outgroup (Lepus crawshayi). (TIF) [file pone.0037069.s001.tif]
