# Supplementary material for: Testing the Fitness Consequences of the Thermoregulatory and Parental Care Models for the Origin of Endothermy
Source: PLoS One. 2012 May 14;7(5):e37069. doi: 10.1371/journal.pone.0037069 (PMC3351390; doi:10.1371/journal.pone.0037069)
Supplement: Table S1 — Alphabetical listing of metabolic and population data compiled for 17 species of rodents with references. (DOCX) [file pone.0037069.s002.docx]

Table S1. Alphabetical listing of metabolic and population data compiled for 17 species of rodents with references.

| Species | MMR  (mlO_2_/h) | BMR  (mlO_2_/h) | Tb  (ºC) | Mb (g) | *R_max_* | Source(s) |
| --- | --- | --- | --- | --- | --- | --- |
| *Apodemus flavicollis* | 300.4 | 75.1 | 36.6 | 25.9 | 2.462 | 4,17 |
| *Baiomys taylori* | 84.9 | 19.7 | 37.0 | 6.9 | 0.978 | 5,21 |
| *Akodon olivaceus* | 248.4 | 49.4 | 37.2 | 27.0 | 1.574 | 1,2,3 |
| *Clethrionomis rutilus* | 378.0 | 77.0 | 37.4 | 28.0 | 0.972 | 8,22 |
| *Dipodomys merriami* | 290.9 | 59.5 | 34.1 | 36.5 | 1.871 | 10,7 |
| *Ellobius talpinus* | 279.6 | 94.0 | 36.9 | 43.7 | 0.583 | 12, 23 |
| *Notomys alexis* | 208.7 | 49.7 | 37.9 | 38.8 | 0.984 | 9,19 |
| *Octodon degus* | 1072.5 | 181.4 | 37.2 | 195 | 1.748 | 15,24 |
| *Peromyscus maniculatus* | 130.9 | 31.3 | 36.3 | 13.3 | 0.229 | 10,24 |
| *Phyllotis darwini* | 407.1 | 71.4 | 36.2 | 59.0 | 2.195 | 1,2,13 |
| *Rattus coletti* | 691.0 | 122.6 | 36.2 | 165.7 | 1.372 | 9,14 |
| *Spermophilus beldingi* | 1856.9 | 233.9 | 38.5 | 303.8 | 1.356 | 18,16 |
| *Uromys caudimaculatus* | 2658.3 | 573.3 | 34.6 | 819.0 | 1.920 | 9,19 |
| *Rattus vilossisimus* | 869. 2 | 149.5 | 35.9 | 253.4 | 0.963 | 9,14,19 |
| *Peromyscus eremicu* | 144.4 | 26.9 | 36.1 | 19.1 | 0.734 | 11, 24 |
| *Perognathus fallax* | 166.6 | 27.8 | 32.6 | 21.2 | 1.021 | 11,24 |
| *Pseudomys gracilicaudatus* | 327.0 | 87.6 | 36.8 | 80.4 | 1.540 | 6,2 |

1.- Bozinovic and Rosenman (1988); 2.- Bozinovic and Rosenman (1989); 3.- Lima et al. (2006); 4.-Cygan (1985); 5.- Rosenmann and Morrison (1974); 6.- Dawson and Dawson (1981); 7.- Lima et al. (2008); 8.- Rosenmann et al. (1975); 9.- Hinds et al. (1993); 10.- Hinds and Rice-Warner (1992); 11.- Hulbert et a. (1985); 12.- Moshkin et al. (2001); 13.- Crespin and Lima (2006); 14.- Madsen and Shine (2002); 15.- Rosenmann (1977); 16.-Sherman and Morton (1984); 17.- Pucek et al. (1993); 18.- Chappel and Bachman (1995); 19.- Predavec and Dickman (1993); 20.- Morris et al. (2000); 21.- Grant et al. (1985); 22.- West (1982); 23.- Evdokimov (2003) and 24.- Global Population Dynamics DataBase (http://www3.imperial.ac.uk/cpb/research/patternsandprocesses/gpdd).

**References Table S1**

Bozinovic, F., and M. Rosenman. 1988. Comparative energetic of South American cricetid rodents. Comparative Biochemistry and Physiology A 91: 195-202.

Bozinovic, F., and M. Rosenman. 1989. Maximum metabolic rate of rodents: physiological and ecological consequences on distributional limits. Functional Ecology 3:173-181.

Chappel, M. A., and G. C. Bachman. 1995. Aerobic performance in Belding’s ground squirrel: variance, ontogeny, and the aerobic capacity model of endothermy. Physiological Zoology 68: 421-442.

Crespin, L., and M. Lima. 2006. Supervivencia adulta y dinámica poblacional del lauchón orejudo *Phyllotis darwini* en Chile Central. Revista Chilena de Historia Natural 79:295-308.

Cygan, T. 1985. Seasonal changes in thermoregulation and maximum metabolism in the yellow-necked field mouse. Acta Theriologica 30:115-130.

Dawson, T. J., and W. R. Dawson. 1982. Metabolic scope and conductance in response to cold of some dasyurid marsupials and Australian rodents. Comparative Biochemistry and Physiology Part A: Physiology 71:59-64.

Evdokimov, N. G. 2003. Fluctuations of population size and structure in the Northern Mole Vole: preliminary analysis. Russian Journal of Ecology 34: 202-209.

Grant, W. E., P. E. Carothers, and L. A. Gidley. 1985. Small mammal community structure in the Postoak Savanna of East-Central Texas. Journal of Mammalogy 66: 589-594.

Hinds, D., and C. N. Rice-Warner. 1992. Maximum metabolism and aerobic capacity in heteromyids and other rodents. Physiological Zoology 65: 188-214.

Hinds, D., R. V. Baudinette, R. E. MacMillen and E. A. Halperns. 1993. Maximum metabolism and the aerobic factorial scope of endotherms. Journal of Experimental Biology 182: 41-56.

Hulbert, A. J., D. S. Hinds, and R. E. MacMillen. 1985. Minimal metabolism, summit metabolism and plasma thyroxine in rodents from different environments. Comparative Biochemistry and Physiology A 81: 687-693.

Lima M., M. A. Previtali, and P. L. Meserve. 2006. Climate and small rodent dynamics in semi-arid Chile: the role of lateral and vertical perturbations and intra-specific processes. Climate Research 30: 125-132.

Lima, M., S. K. M. Ernest, J. H. Brown, A. Belgrano, and N. C. Stenseth. 2008. Chihuahuan desert kangaroo rats: nonlinear effects of population dynamics, competition, and rainfall. Ecology 89 (9): 2594-2603.

Madsen, T., and R. Shine. 2002. Rainfall rats: Climatically-driven dynamics of a tropical rodent population. Australian Journal of Ecology 24: 80-89.

Morris, D. W., B. J. Fox, J. Lou, V. Monamy. 2000. Habitat dependent competition and the coexistence of Australian Heathland rodents. Oikos 91: 294-306.

Moshkin, M. P., E. A. Novikov, and D. V. Petrovski. 2001. Seasonal changes of thermoregulation in the mole vole *Ellobius talpinus*. Physiological and Biochemical Zoology 74: 869-875.

Predavec, M., and C. R. Dickman. 1993. Populations dynamics and habitat use of the long-haired rat (*Rattus villosissimus*) in South-western Queensland. Wildlife Research 21: 1-10.

Rosenmann, M., and P. R. Morrison. 1974. Maximum oxygen consumption and heat loss facilitation in small homeotherms by He-O_2_. American Journal of Physiology 226: 490-495.

Rosenmann, M., P. R. Morrison, and D. Feist. 1975. Seasonal changes in the metabolic capacity of read-blacked voles. Physiological Zoology 48: 303-310.

Rosenmann, M. 1977. Regulacion termica en *Octodons degus*. Medio Ambiente 3: 127-131.

Sherman, P. W., and M. L. Morton. 1984. Demography of Belding’s ground squirrels. Ecology 65: 1617-1628.

West, S. D. 1982. Dynamics of colonization and abundance in Central Alaskan populations of the Northern red-backed vole, *Clethrionomys rutilus*. Journal of Mammalogy 63: 128-143.
