# Supplementary material for: Testing the Fitness Consequences of the Thermoregulatory and Parental Care Models for the Origin of Endothermy
Source: PLoS One. 2012 May 14;7(5):e37069. doi: 10.1371/journal.pone.0037069 (PMC3351390; doi:10.1371/journal.pone.0037069)
Supplement: Table S2 — IRBP gene sequences used in phylogenetic reconstruction and its GenBank code. (DOCX) [file pone.0037069.s003.docx]

Table S2. IRBP gene sequences used in phylogenetic reconstruction and its GenBank code.

| Family | Species | Specie's sequence | Code GenBank |
| --- | --- | --- | --- |
| Muridae | *Apodemus flavicollis* | *Apodemus mystacinus* | AB303229.1 |
| Muridae | *Uromys caudimaculatus* | *Uromys caudimaculatus* | EU349875.1 |
| Muridae | *Rattus coletti* | *Ratus exulans* | AY326105.1 |
| Muridae | *Rattus vilossisimus* | *Ratus tanezunami* | DQ191515.1 |
| Muridae | *Pseudomys gracilicaudatus* | *Pseudomys australis* | AM910939.1 |
| Muridae | *Notomys alexis* | *Mastacomys fuscus* | EU349856.1 |
| Cricetidae | *Ellobius talpinus* | *Microtus socialis* | FM162055.1 |
| Cricetidae | *Chlethrionomys rutilus* | *Chletrionomys gapperi* | AY326080.1 |
| Cricetidae | *Baiomys taylori* | *Baiomys taylori* | EF989837.1 |
| Cricetidae | *Peromyscus maniculatus* | *Peromyscus maniculatus* | EF989884.1 |
| Cricetidae | *Peromyscus eremicus* | *Peromyscus eremicus* | EF.989877.1 |
| Cricetidae | *Akodon olivaceus* | *Abrothrix andinus* | AY277418.1 |
| Cricetidae | *Phyllotis darwini* | *Phyllotis xanthopygus* | AY277471.2 |
| Octodontidae | *Octodon degus* | *Octodon lunatus* | AM050863.1 |
| Sciuridae | *Spermophilus beldingi* | *Spermophilus tridecemaculatus* | AF297278.1 |
| Heteromydae | *Perognathus fallax* | *Perognathus flavus* | GQ480822.1 |
| Heteromydae | *Dipodomys merriami* | *Dipodomys merriami* | AJ427233.1 |
